# Short-term follow-up HRCT Chest of COVID-19 survivors and association with persistent dyspnea

**DOI:** 10.1186/s43055-021-00607-w

**Published:** 2021-09-21

**Authors:** Ishan Kumar, Adity Prakash, Manoj Ranjan, Sankha Shubhra Chakrabarti, Ram C. Shukla, Ashish Verma

**Affiliations:** 1 Departments of Radiodiagnosis, Institute of Medical Sciences, Banaras Hindu University, Varanasi, 221005 India; 2 Departments of Radiodiagnosis, Heritage Institute of Medical Sciences and Heritage Hospital, Varanasi, India; 3 Departments of Geriatrics, Institute of Medical Sciences, Banaras Hindu University, Varanasi, India

**Keywords:** COVID 19, Coronavirus, HRCT, Fibrosis, Interstitial lung disease, Post-COVID sequelae

## Abstract

**Background:**

There is an increasing concern that a proportion of the survivors of COVID 19 might develop fibrotic and/or other non-reversible lung changes. The aim of this retrospective study was to review the imaging findings of HRCT of lungs in a cohort of COVID 19 survivors, coming for short-term clinical follow-up and to assess the relation of the observed HRCT changes with the presence of dyspnea.

**Results:**

In total, 40 patients with residual CT findings were included in this study with a mean age of 44.3 years and male: female ratio of 3:2. The presence of residual ground-glass opacities (85%) and reticular opacities (80%) was the most common findings. 25% of the cases had cystic changes in their lung. The presence of dyspnea was significantly associated with male sex and a history of smoking. On HRCT, the presence of cystic changes, involvement of > 10 lung segments, and an HRCT severity score > 7 were significantly associated with dyspnea.

**Conclusion:**

Survivors of COVID 19 demonstrate persistent changes in the lung on HRCT. We recommend that a follow-up HRCT should be performed in these patients to identify those with post-COVID sequelae.

## Background

Many countries have started experiencing the second wave of the infection while many are still fighting the first wave. While most of the survivors are doing well after their recovery, a substantial minority continues to have respiratory symptoms such as dyspnea and fatigue. There is increasing concern that a proportion of the survivors of COVID 19 might develop fibrotic and/or other non-reversible lung changes, either due to scarred pulmonary parenchyma or due to ventilator-induced damage [1, 2]. This study aimed to review the imaging findings of HRCT of lungs in the COVID 19 survivors, coming for short-term clinical follow-up and to assess the relation of the observed HRCT changes with the presence of dyspnea.

## Methods

### Subjects

This was a retrospective study, conducted in a University-based tertiary care Hospital approved by the Institutional ethical committee of Institute of Medical Sciences, Banaras Hindu University. Informed consent was obtained from all the patients included in the study. The inclusion criteria of the study were patients who earlier tested COVID-19 positive on RT-PCR and were subsequently discharged as per the discharge criteria and came for follow-up CT scan with or without any respiratory symptoms. Patients with normal CT scans in the follow-up period were excluded from this study. The discharge criteria of our institute for COVID-19 patients were (a) 10 days of onset of symptoms and absence of fever for 3 days for mild COVID cases, (b) clinical recovery and ability to maintain oxygen saturation for 3 days without support for three days for moderate COVID, (c) clinical resolution and subsequent negative RT PCR test.

Various clinical data such as the age of the patient, gender, clinical symptoms, presence of co-morbidities, respiratory rate, and presence or absence of persistent dyspnea at the follow-up period were collected.

### HRCT chest protocol and interpretation

Chest CT images of these patients were obtained on a GE light Speed VCT 128 slice CT scanner. The patient was placed in a supine position. The scanning parameters were kV = 120, 40–50 mAs, pitch 0.99–1.22 mm, matrix 512 × 512, slice thickness 1.25 mm.

The CT chest features were analyzed by two radiologists in tandem with 9 and 17 years of experience. Various lesions such as the presence of ground-glass opacities, consolidations, reticulations, traction bronchiectasis, cystic changes, nodules, lymphadenopathy, pleural thickening, pleural or pericardial effusion, and pneumo-mediastinum were assessed. The number of segments affected out of a total of 18 segments of bilateral lungs was counted. The predominant pattern of opacity was categorized as 1. Ground-glass opacities 2. Reticular 3. Cystic. The predominant pattern selection was based on which opacity was most striking and distinct. A semi-quantitative HRCT score was calculated based on the extent of lobar involvement (0: 0%; 1, < 5%; 2:5–25%; 3:26–50%; 4:51–75%; 5, > 75%; range 0–5) for each of the 5 lobes with a global score 0–25, similar to that done in active COVID 19 cases.

### Statistical analysis

All statistical analyses were performed using the automated software SPSS version 18.0. Continuous variables were recorded as mean ± standard deviation, and categorical variables were recorded as frequency and percentages. Fisher's exact test was done for comparing the data between two groups. HRCT findings were compared between the groups with and without dyspnea.

## Results

Following the inclusion criteria, the CT of 84 patients was analyzed. Out of these, 44 patients had a normal CT of the chest and were excluded from the study. The remaining 40 patients with residual CT findings were included in this study. The mean duration of interval between follow-up CT scan and discharge was 48 days (range 10th–84th day). The mean age of these patients was 44.3 years with a male: female ratio of 3:2. The mean age of patients with dyspnea was 50.2 ± 13.1 years, whereas the mean age of patients without dyspnea was 39.5 ± 18.5 years.

**Fig. 1:**
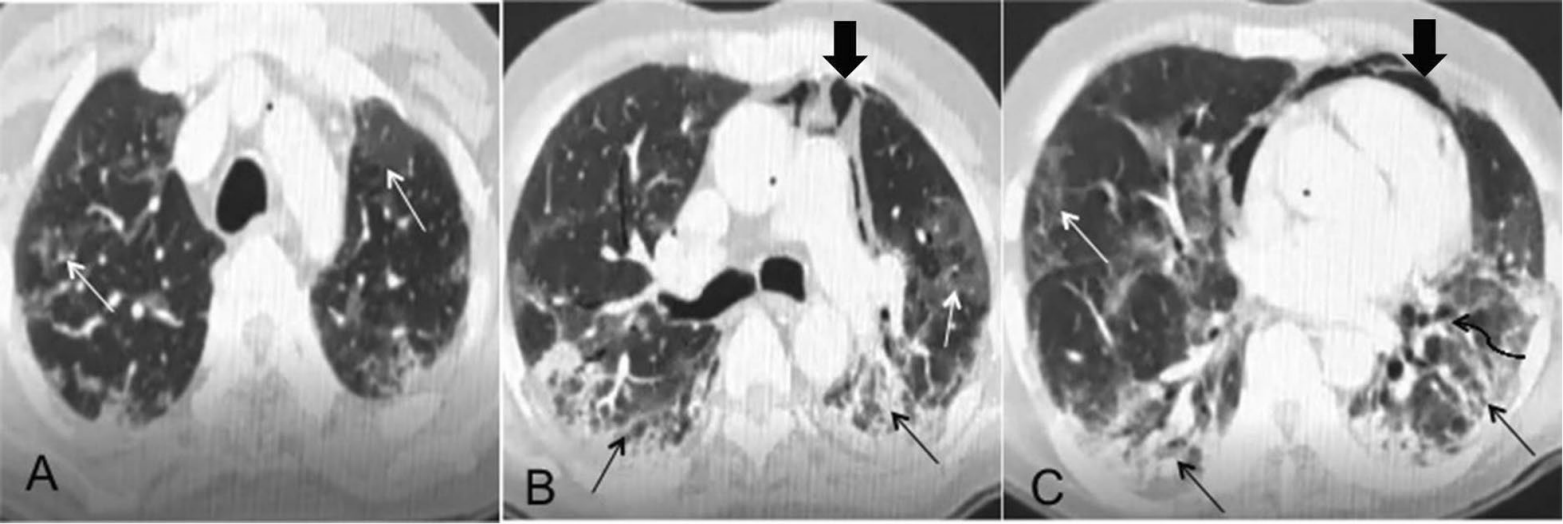
Reticular predominant changes. Bilateral lungs show predominant reticular changes with interlobular septal thickening, fibrotic bands, and subpleural fibrotic changes similar to that observed in usual interstitial pneumonia (black arrows). Also notes are patchy areas of ground-glass opacities (white arrows) in bilateral lungs and tractional bronchiectasis (black curved arrow). Pneumomediastinum is also seen (solid black arrow)

**Fig. 2:**
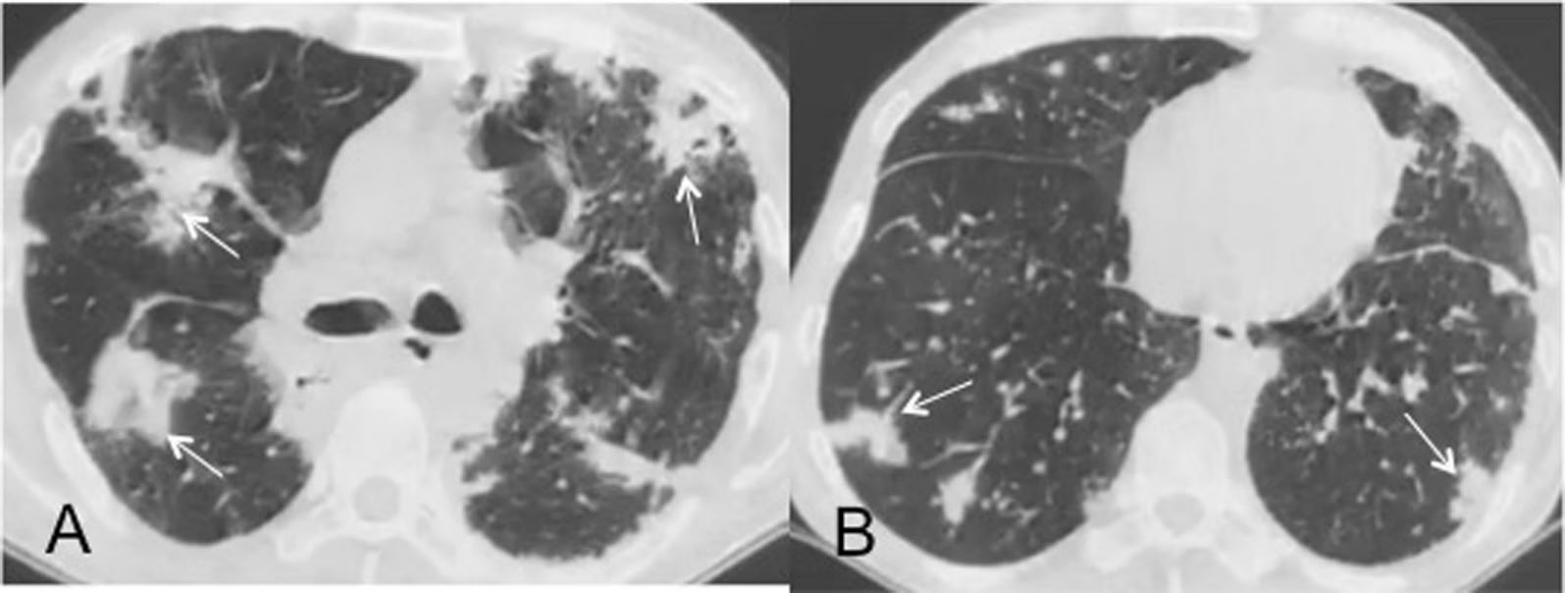
Bilateral confluent fibrotic changes with consolidations (white arrows) in a patient 21 days after discharge

**Fig. 3:**
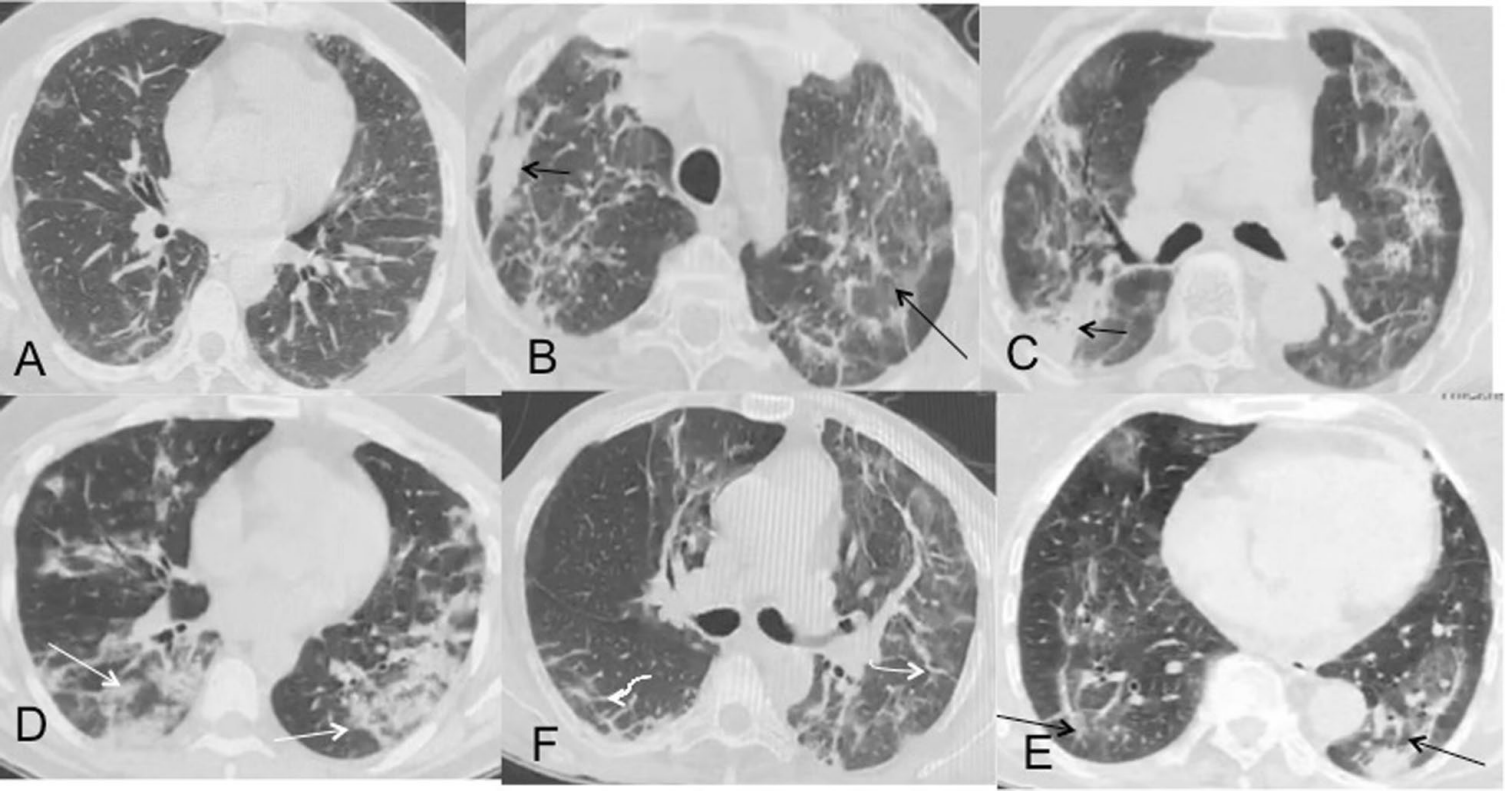
Spectrum of HRCT changes in patients with reticular predominant post-COVID sequelae. **a** Mild reticular changes with just subpleural fibrotic stripes in posterior basal segments of bilateral lower lobes, **b** reticular opacities in both lungs with confluent fibrosis (black arrow) and atoll sign (long black arrow) **, c** reticular opacities with consolidations (black arrow), **d** reticulations with consolidations (white long arrows), **e** reticulations with bilateral interlobular septal thickening (curved arrows), **f** bilateral reversed halo (atoll) sign (long black arrows)

Table 1 shows the summary of HRCT findings at the follow-up period. Diffuse involvement (65%) and a combination of central and peripheral lesions (75%) were most common in follow-up scans. Notably, we did not find any case where the lesions were limited to central/perihilar lung fields. The presence of residual ground-glass opacities (85%) and reticular opacities (80%) was the most common findings (Fig. 1). 25% of the cases had cystic changes in their lung. Also, 35% of cases had tractional bronchiectasis, due to the fibrotic adjacent changes. Patchy consolidations were found in 8 cases (Fig. 2). The predominant pattern of involvement was reticular and fibrotic in 55% cases (Fig. 3), diffuse or multifocal ground-glass opacity in 25% cases, and cystic disease in 20% cases.

**Table 1:** Summary of CT findings and their association with dyspnea

Radiological findings	Frequency (%)	Dyspnea		*P* value
		Present (n = 18)	Absent (n = 22)	
Ground-glass opacity	34 (85%)	14	20	0.56
Consolidations	8 (20%)	6	2	0.28
Reticulation	32 (80%)	14	18	1
Traction bronchiectasis	14 (35%)	8	6	0.64
Cystic changes	10 (25%)	10	0	0.026
Sub- pleural stripes	16 (40%)	10	6	0.36
Fibro- parenchymal bands	20 (50%)	6	14	0.37
*Distribution*				
Central	0 (0%)	0	0	0.32
Peripheral	10 (25%)	2	8	
Central and peripheral	30 (75%)	16	14	
Patchy	14 (35%)	0	14	0.001
Diffuse	26 (65%)	20	6	
*No. of segments involved*				
< 10 Segments	14(35%)	0	14	0.005
> = 10 segments	26 (65%)	18	8	
*HRCT severity score*				
< = 7	10	0	10	0.038
> 7	30	18	12	
*Overall Predominant CT pattern*				
Reticular pattern	22 (55%)	8	14	
Cystic pattern	8 (20%)	6	2	
Ground- glass pattern	10 (25%)	4	6

**Fig. 4:**
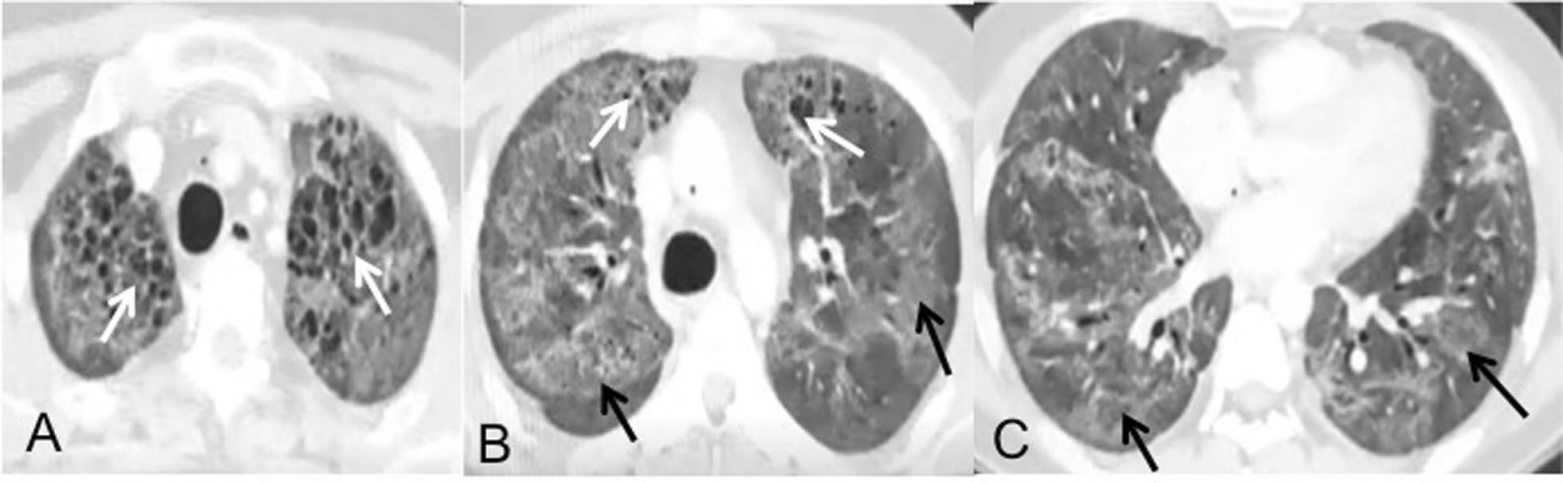
Bilateral lungs show diffuse ground-glass opacities (black arrows) with diffusely interspersed microcystic changes predominantly located in upper lobes (white arrows)

**Fig. 5:**
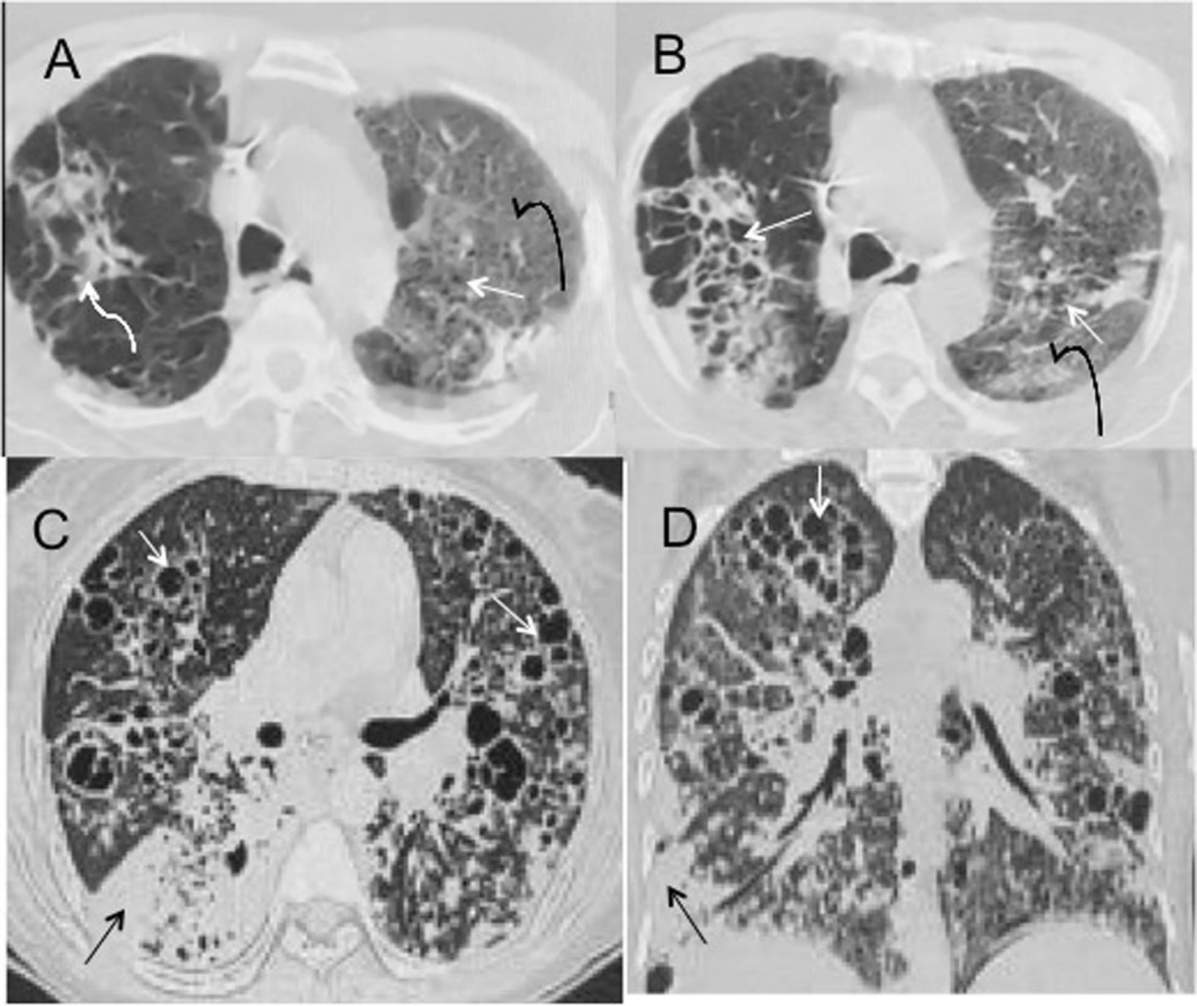
Cystic changes as post-COVID sequelae. **a** Reticular changes in right upper lobe (curved white arrows) with GGO in left upper lobe (curved black arrow) with interspersed microcystic changes (white arrow), **b** honeycombing like cystic changes with adjacent reticulations (white arrow) with GGO in left upper lobe (curved black arrow), **c**, **d** axial and coronal image of a patient showing extensive bilateral cystic lung disease (white arrows) with consolidation in right lower lobe (black arrow) due to superimposed infection

Cystic changes in the lungs were associated with diffuse GGO in three cases and resembled the appearance of microcystic non-specific interstitial pneumonitis (Fig. 4). In four cases, the cystic changes were associated with adjacent reticulations similar to honeycombing observed in idiopathic pulmonary fibrosis. In one case, the entire lung was replaced by multiple cysts of variable sizes (Fig. 5). GGOs predominant lungs were seen either diffuse GGO occurring in both lungs or as multifocal patchy lesions. In two of the cases, we observed that GGO was seen replacing the consolidation patches present during the active COVID course and there was an increase in the involved area compared to previous CT. Four of the cases in our study showed "atoll sign" in the follow-up HRCT with central GGO surrounded by an incomplete rim of fibrosis (Fig. 3).

Table 2 shows the association of dyspnea with patients' age, sex, and coexisting illnesses. Out of 40 cases in with radiological follow-up, 18 patients presented with persisting dyspnea in the follow-up period. The presence of dyspnea was significantly associated with male sex and a history of smoking. Age, history of systemic hypertension, diabetes, and history of other chronic illnesses had no significant association with the presence of dyspnea at follow-up. Dyspnea was absent in 12/14 (85%) of patients with age < 40 years and was present in 61% of patients with age > 40 years. The incidence of dyspnea was significantly higher in males (66%) compared to females (12.5%). 18 patients had a past history of chronic cigarette smoking of which 16 (89%) had dyspnea which was significantly higher than non-smokers (9%). The incidence of dyspnea was similar in patients with or without diabetes, hypertension, and other chronic illnesses.

**Table 2:** Association of dyspnea with patients age, sex, and coexisting illnesses

Parameters	Dyspnea present	Dyspnea absent	*P* value
Age < 40	2	12	0.07
Age > 40	16	10	
Males	16	8	0.02
Females	2	14	
*Smoker*	16	2	< 0.001
Non- smoker	2	20	
Hypertensive	10	8	0.65
Non- hypertensive	8	14	
Diabetic	0	10	0.53
Non- diabetic	8	22	
Chronic disease	4	0	0.52
No history of chronic disease	22	14	

Table 1 shows the association of dyspnea with HRCT findings. Of the various radiological findings present in the follow-up CT of COVID-19 patients, the presence of cystic changes was significantly associated with dyspnea with 100% of the patients having cystic changes having dyspnea. Ground-glass opacities, consolidations, reticular opacities, bronchiectasis, fibrotic bands, and subpleural stripes were similarly distributed between patients with or without dyspnea. Distribution of the CT findings had a more significant impact on the presence of dyspnea. The involvement of 10 or more segments was significantly associated with dyspnea (69%) while none of the patients with involvement < 10 segments had dyspnea.

Ten patients with HRCT severity score graded as mild were not associated with dyspnea in the follow-up period.

Table 3 shows the temporal association of HRCT findings with duration since discharge; 20 patients presented within 1 month of discharge. HRCT findings such as tractional bronchiectasis and cystic changes were more common in patients who presented after one month of discharge, whereas consolidations, subpleural fibrous stripes were more common in early presentations. The involvement of > 10 segments was more common in the early (< 1 month) presentation. Presence of GGO, reticulations were similarly distributed in both groups.

**Table 3:** Temporal association of HRCT findings with duration since discharge

Features	Duration since discharge < = 1 month (*n* = 20)	Duration since discharge > 1 month (*n* = 20)
Peripheral opacities (*n* = 10)	2	8
Both peripheral and central opacities (*n* = 30)	18	12
Patchy opacities (*n* = 14)	2	12
Diffuse opacities (*n* = 26)	18	8
Ground- glass opacity (*n* = 34)	16	18
Consolidations (*n* = 8)	6	2
Reticulations (*n* = 32)	18	14
Traction bronchiectasis (*n* = 14)	4	10
Cystic changes (*n* = 10)	2	8
Sub- pleural fibrous stripes (*n* = 16)	12	4
Sub-pleural sparing (*n* = 6)	4	2
Fibroparenchymal bands (*n* = 20)	8	12
No. of segments affected < = 10 (*n* = 14)	2	12
No. of segments affected > 10 (*n* = 26)	18	8
Reticular pattern (*n* = 22)	14	8
Ground-glass pattern (*n* = 10)	4	6
Cystic pattern (*n* = 8)	2	6

## Discussion

Most of the research in COVID 19 so far has focused on early diagnosis, identifying the pathophysiology of the infection and treatment of the acute illness aiming to reduce mortality and shortening hospital stay. With a constantly increasing population of COVID 19 survivors, clinicians are increasingly getting cognizant of the fact that many of the patients experience persistent respiratory symptoms. Attention is now shifting toward the follow-up of COVID 19 survivors and the identification of any long-term sequelae [3].

A study performed on 110 survivors of a global outbreak of Severe acute respiratory syndrome (SARS) in 2003 revealed significant impairment of exercise capacity and surface area for gas exchange in as many as 15.5% of survivors [4]. Residual HRCT findings such as GGO, coarse reticular opacities, intralobular and interlobular septal thickening, predominantly in anterior lung segments were persistently noted years after recovery from SARS [5, 6]. Predicated on the semblances between ongoing (COVID 19) and previous SARS coronavirus infections, and a few of the isolated case reports, there is speculation of significant fibrotic lung disease that might result in functional disabilities in the among survivors [7]. HRCT, in combination with pulmonary function tests, can be an irreplaceable tool to evaluate the COVID-19 survivors and to elucidate the long-term effect. Moreover, the early recognition of the fibrotic and other lung changes on CT and the categorization of the accompanying risk can prompt the initiation of antifibrotic treatment to prevent further progression. [8, 9].

Our study showed that HRCT can successfully demonstrate residual lesions in the lungs of COVID survivors. Also, the characterization of patterns and extent of the involvement on HRCT can help in the recognition of the patients who are predisposed toward developing clinically relevant pulmonary dysfunction.

Isolated case reports and few studies have shown various HRCT changes in the survivors of COVID 19. A study by Liu et al. who found interlobular septal thickening, fibrous stripes, GGO, and subpleural lines in the early follow-up period [10]. In our study, the majority (55%) of cases presented with reticular and fibrotic predominant changes while diffuse or multifocal GGO was the predominant finding in 25% of cases. Also, we found that 20% of patients presented with predominant cystic changes, a finding that has not been reported in previous studies. Cystic changes were found in 10 patients in our study and were the predominant pattern in eight of them. Cystic changes in our cases were either in the background of GGO similar to the changes in microcystic NSIP or within the adjacent reticular fibrotic changes similar to honeycombing in IPF. One patient showed cystic changes resembling primary cystic diseases of lungs, like cystic fibrosis. These changes have not been described in the existing radiological literature on COVID 19.

Fibrotic changes in our cases were seen as diffuse or multifocal reticulations (interlobular and intralobular septal thickening, fibrotic parenchymal bands, subpleural stripes, and tractional bronchiectasis in our study). The imaging findings of fibrotic predominant changes resembled that of idiopathic pulmonary fibrosis. In four cases, GGO with surrounding incomplete ring of fibrosis is similar to the atoll sign observed in organizing pneumonia. The predominance of interstitial lung changes can be attributed to the fact that SARS-CoV-2 uses angiotensin-2-converting enzyme (ACE2) as a receptor causing interstitial lung damage at first before involving lung parenchyma [11]. Studies have found a similar cytokine profile in idiopathic pulmonary fibrosis and COVID 19 suggesting similar histopathological processes and explaining similar HRCT findings [12]. Grillo et al. have performed cryobiopsy in eight patients who died of COVID 19 and observed marked fibrosis, characterized by fibroblast proliferation, airspace damage, and micro- honeycombing [13]. The cause of fibrosis may be virus-induced changes, ventilation associated lung injury, or sequelae to acute respiratory distress syndrome as 85% of patients with ARDS show anterior reticulation on HRCT and experience long-term pulmonary function abnormality [9].

Tractional bronchiectasis and cystic changes were more common in patients with > 1-month presentation, whereas patchy consolidations, subpleural fibrous stripes were more common in early (< 1 month) presentations. This might be attributed to the fact that the lesions were not fully resorbed in patients who presented earlier than 1 months for follow-up. This was further supported by the observation that the involvement of > 10 segments was more common in early (< 1 month) presentation. GGO was seen as a tinted sign which is a dispersion of the opacities with an increase in the overall area of involvement and a decrease in the degree of opacity. This might be due to the re-expansion of alveoli and the resolution of inflammation. A study by Liu et al. evaluating short-term changes in discharged patients of COVID 19 has observed replacement of GGO with fibrotic patches which eventually are resorbed or show reduced density [10]. GGO occurring in combination with fibrotic changes can also represent non-specific interstitial pneumonia.

Few studies have shown that HRCT findings during the time of hospitalization such as the presence of fibrotic parenchymal bands and irregular pleuro-pulmonary interface and involvement of more segments can predict the formation of pulmonary fibrosis [14]. Moreover, the development of pulmonary fibrosis also is related to the severity of their acute COVID 19 course with complete resolution in patients with a mild course [3]. Unfortunately, we could not compare the follow-up CT with the CT obtained at the time of active COVID infection because many of the patients in our study did not undergo HRCT at the time of active infection.

In our cohort, dyspnea was significantly associated with male gender and previous history of smoking, whereas higher age, presence of diabetes, hypertension, and other chronic illnesses were not significantly associated with dyspnea in the follow-up period. This was in contrast with the previous study by Yu et al. who observed that dyspnea is more likely to be present in elderly or immunocompromised groups [14]. The involvement of < 10 segments in the follow-up was not associated with dyspnea in any of the cases. Moreover, the CT severity score was calculated using the same method as is done in the active COVID course and a score of 7 or less was not associated with dyspnea in any of the cases. This information is of importance to assign the extent of involvement that can translate into clinical breathlessness. Of the various CT findings, the presence of predominant cystic changes was associated with dyspnea. A study by Yu et al. showed that fibrotic changes in follow-up CT were significantly associated with dyspnea and higher respiratory rate compared to those in the non-fibrosis group [14].

Our study had a few limitations. Firstly, we have a small sample size as cases from a single centre were included. The second limitation was a small duration of follow-up. A longer follow-up would have enabled us to evaluate whether the changes observed at short-term follow-up were eventually resolved or were persistent. Moreover, we could not compare the HRCT findings of all the patients during and after their infection as many of these patients did not undergo HRCT during their active infection course. Furthermore, the complete clinical assessment of the patients in their follow-up could not be done owing to the current pandemic situation. A comprehensive clinical assessment including spirometry, carbon monoxide transfer factor, inspiratory and expiratory respiratory muscle strength, 6-min walk distance would have probed a more meaningful correlation with HRCT findings.

## Conclusions

To conclude, our study showed that involvement of > 10 segments of the lung, HRCT severity score of > 7, and presence of cystic changes in lungs of COVID 19 survivors seems to be associated with post-COVID persistent dyspnea. This was one of the initial studies attempting to characterize the HRCT findings that could translate into morbidity associated with post-COVID sequelae. Due to our small sample size, HRCT changes reported here may not representative of all patients who survived COVID-19 and larger multicentric studies should be the next step in tackling the COVID-19 pandemic.

## Data Availability

The datasets analyzed during the current study are available with the corresponding author.

## Abbreviations

HRCT: High-resolution computed tomography.
COVID19: Coronavirus disease 19
RTPCR: Reverse transcription polymerase chain reaction
GGO: Ground-glass opacities
SARS: Severe acute respiratory syndrome
